# Exploring Field-Induced Fragmentation of Protonated Alcohols: Mechanistic Insights and Stabilizing Ion–Solvent Clusters

**DOI:** 10.1021/jasms.5c00348

**Published:** 2025-12-15

**Authors:** Philip Timmermann, Anjita G C Paudel, Gary Eiceman, Stefan Zimmermann, Alexander Haack

**Affiliations:** † Department of Sensors and Measurement Technology, Institute of Electrical Engineering and Measurement Technology, Leibniz University Hannover, 30167 Hannover, Germany; ‡ Department of Chemistry and Biochemistry, New Mexico State University, Las Cruces, New Mexico 88003, United States

**Keywords:** Ion Fragmentation Mechanism, Ion Heating, Ion Mobility, Density Functional Theory, Transition State Theory

## Abstract

Field-induced ion activation in medium to high pressure regions of a mass spectrometer or ion mobility spectrometer can lead to changes in the ion structure, namely unfolding, tautomerization, or fragmentation. To either prevent mislabeling of spectra or utilize these effects efficiently, the underlying ion dynamics need to be understood. Hydroxyl-containing compounds in particular show significant fragmentation (loss of H_2_O), yet the energetics and mechanisms are not well studied. This is particularly true for primary hydroxyl groups, as the presumably formed primary carbocations are highly instable. In this study, we investigate the dynamics of the field-induced fragmentation of protonated primary and secondary alcohols using a combined theoretical and experimental approach. Specifically, we combine density functional theory and reaction kinetics modeling with fragmentation measurements using a HiKE-IMS-MS and tandem IMS device. We find that the fragmentation mechanism of both primary and secondary protonated alcohols proceeds via a protonated cyclopropane (PCP^+^) moiety. Especially for primary alcohols, this moiety enables an intramolecular S_N_2 reaction where the neutral H_2_O at the terminal carbon is substituted by an H-shift, directly yielding a secondary carbocation. Our results suggest quite high fragmentation rates, even at moderate ion activations, rendering protonated alcohols very unstable. However, we also find that neutral background water can form ion–solvent clusters with the protonated alcohols that effectively prevent the fragmentation. This could also help stabilize other labile ions in the future.

## Graphical abstract

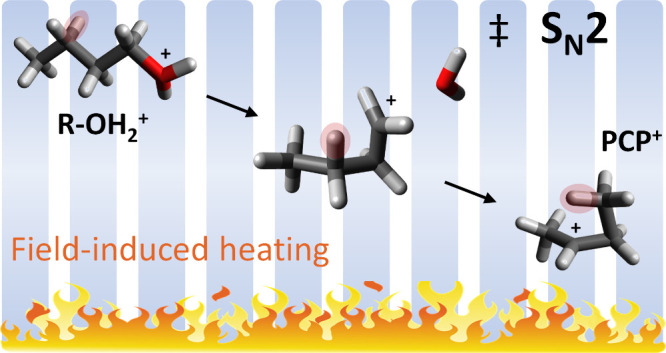

## Introduction

The manipulation of ions via electric fields in high to medium pressure regions of a mass spectrometer (MS), for example, in the ion source, ion guides, radio frequency (RF) funnels, or ion mobility spectrometry (IMS) devices, can significantly alter their internal energy (ion activation) and can lead to structural changes like unfolding, tautomerization, or fragmentation.^1^ These transformations can lead to misannotation of spectra: unfolding or tautomerization can alter the ion’s collision cross section (CCS) as measured by IMS, while fragmentation changes the ion’s mass (as well as CCS).^2–4^ Conversely, ion activation and the resulting transformations can also be used to harness additional information about the analyte studied. For example, fragmentation is widely used to study molecular composition and thus increase selectivity, including as collision-induced dissociation (CID) in tandem MS,^5^ sometimes in combination with ion–molecule chemistry or spectroscopic techniques,^6^ as deliberately triggered in-source fragmentation,^7^ or as field-induced fragmentation in IMS devices.^8–10^ Especially as more and more complex samples are studied in fields like metabolomics and lipidomics, there is a growing need for a thorough understanding of the dynamics, i.e., the mechanisms and energy dependencies of these processes, either to avoid (or at least anticipate) unwanted transformation or to efficiently use them.^1,3,11–13^

One functional group that is ubiquitously present in many analytes in pharmacology and biochemistry and often subject to fragmentation is the hydroxy (R–OH) group. Due to its significant proton affinity, it is readily protonated in positive ionization, which leads to a weakening of the R-O bond. Indeed, fragmentation of analytes with protonated hydroxy groups, i.e., the loss of water (R–OH_2_^+^ → R^+^ + H_2_O), has been observed in-source for drugs,^14^ steroids,^15,16^ serine- or threonine-containing peptides,^17–19^ underivatized carbohydrates,^20^ carotenoids,^21^ and sphingolipids.^22^ In fact, many studies from proton-transfer reaction MS (PTR-MS),^23,24^ selected ion flow tube MS (SIFT-MS),^25^ IMS-MS couplings,^26,27^ and IMS applying high field strengths^28,29^ have investigated the fragmentation patterns and branching ratios of simple alcohols and often observed loss of water.

Despite all of these observations and studies, many questions remain unanswered. For example, while water loss of *n*-alcohols is readily observed, methanol and ethanol show negligible fragmentation.^23^ Further, while the fragmentation of secondary or tertiary alcohols will yield stable secondary or tertiary carbocations, respectively, loss of water from a primary carbocation would yield an unstable primary carbocation. It has already been speculated that some sort of (quick) rearrangement is necessary, as experiments show high fragmentation yields even for primary alcohols.^25^ Yet, this mechanism has not been identified.

In this study, we investigate the fragmentation dynamics caused by field-induced heating of primary and secondary alcohols (as model systems for hydroxy containing compounds). Namely, we use extensive modeling to investigate rearrangement/fragmentation mechanisms, their energetics and rate constants, and their dependency on the applied reduced field strengths. Here, field-induced heating refers to ion activation by electric fields at high to medium pressures (many collisions), where the ion energy distribution is still characterized by a temperature, albeit a higher temperature than the background gas temperature. This is different from the situation in CID, where few, high-energy collisions yield a strongly nonthermal ion energy distribution. Consequently, we verify our theoretical results by using two IMS devices that allow for efficient ion activation, one High Kinetic Energy IMS (HiKE-IMS),^30^ operated at 14 mbar with a static but high electric field, and one tandem IMS system with a fragmenter region,^8,31^ operated at atmospheric pressure and activating the ions through an oscillating field. Additionally, we investigate the influence of background solvents (often present in the high to medium pressure regions of the instrument from, e.g., an ESI source) on the fragmentation dynamics.

## Methods

### Computational Modeling

Computational modeling of the ion structure, energetics, reaction paths, and field-induced heating was performed as previously described.^32^ Briefly, we use density functional theory, applying the ωB97X-D3­(BJ)/def2-TZVPP^33–37^ level of theory to perform geometry optimization (both minima and transition states) as well as Hessian calculations for thermochemical data. Here, we explicitly treat movement around all dissociating bonds as hindered internal rotations as opposed to vibrations. On the same level of theory, we compute atomic partial charges using the CHELPG scheme^38^ for later use in the mobility/CCS calculations. Improved electronic energies are obtained applying the DLPNO–CCSD­(T)/def2-TZVPP (TightPNO settings)^39,40^ level of theory. Electronic structure calculations are performed using ORCA (v5.0.4).^41,42^ Subsequently, ion geometries and partial charges are then handed over to OpenBabel^43^ for automatic identification of atom classes within the MMFF94 ^44,45^ force field. These data are then passed over to MobCal-MPI 2.0 ^46^ to obtain mobility, collision cross section, and effective temperature data over the desired range of reduced field strengths (0–120 Td) using third order two-temperature theory (2TT).^47^

Tight transition states (TS) are located by first mapping out the reaction path using either potential energy surface scans or the nudged-elastic band (NEB) method^48^ followed by a TS optimization starting form the highest energy point of the found path. Reaction rate coefficients are then modeled via the Eyring equation (tight TSs) or via SACM theory^49^ (loose TSs) and corrected for their pressure dependence according to a Lindemann–Hinshelwood treatment as published recently.^50^ All optimized structures are available in the ioChem-BD database (see Data Availability Statement).

Field-induced reactions are modeled by evaluating the reaction rate coefficients at the effective temperature of the ions, as defined by the applied field strength:

1 $$T_{\mathrm{eff}}=T_{\mathrm{bath}}+\frac{M}{3k_{B}}{\left({\left[K_{0}\right]}_{3}N_{0}\cdot \frac{E}{N}\right)}^{2}\left(1+{\left[\beta_{2\mathrm{TT}}\right]}_{3}\right)$$

Here, *T*_bath_ is the bath gas temperature, *M* is the molecular mass of the bath gas particles, *k*_B_ and *N*_0_ are Boltzmann’s and Loschmidt’s constants, respectively, *E*/*N* is the reduced field strength (field strength, *E*, divided by the bath gas particle density, *N*), *K*_0_ is the ion’s reduced mobility and β_2TT_ is a correction factor from 2TT. The brackets [···]_3_ denote that the field dependency of the containing quantities is modeled in third order of 2TT. The field-dependent values for [*K*_0_]_3_ as well as [β_2TT_]_3_ are shown in Figure S3 of the Supporting Information (SI) for all protonated alcohols studied and also tabulated in the XLSX file of the SI. We find [β_2TT_]_3_ to be usually below 0.015 (absolute). It should be noted that the usage of eq 1 ignores any effects due to the inelasticity of collisions and conformational changes (e.g., unfolding) of ions, both of which continue to be a the topic of active research.^51–54^ While the magnitude of these effects is difficult to assess, generally, the true effective temperature should be lower than the one modeled via eq 1. Inelasticity leads to loss of kinetic energy into the internal degrees of freedom of the bath gas, effectively cooling the ions.^52,54^ Unfolding of ions at higher ion temperatures decreases the ion’s mobility and thus lowers its drift velocity.^51,53^ Thus, we can only expect qualitative agreement between our modeling and the experiments.

Temporal evolution of the ion chemistry over the course of the drift time can be directly modeled by describing each considered reaction with a corresponding differential equation and an associated rate coefficient. This system of coupled rate equation can then be integrated numerically over time, which yields the population of each ion after a given time *t*. Section S5 of the SI as well as ref 32 (which includes an explicit example calculation of this procedure) provide more details. These populations can be compared directly with measured ion populations (see below) if the reaction time is identified with the residence or drift time in the instrument. This pipeline, in principle, allows for dynamic fields as present in modern IMS-instruments and RF-funnels. The overall modeling workflow is depicted in Figure 1.

**Figure 1:**
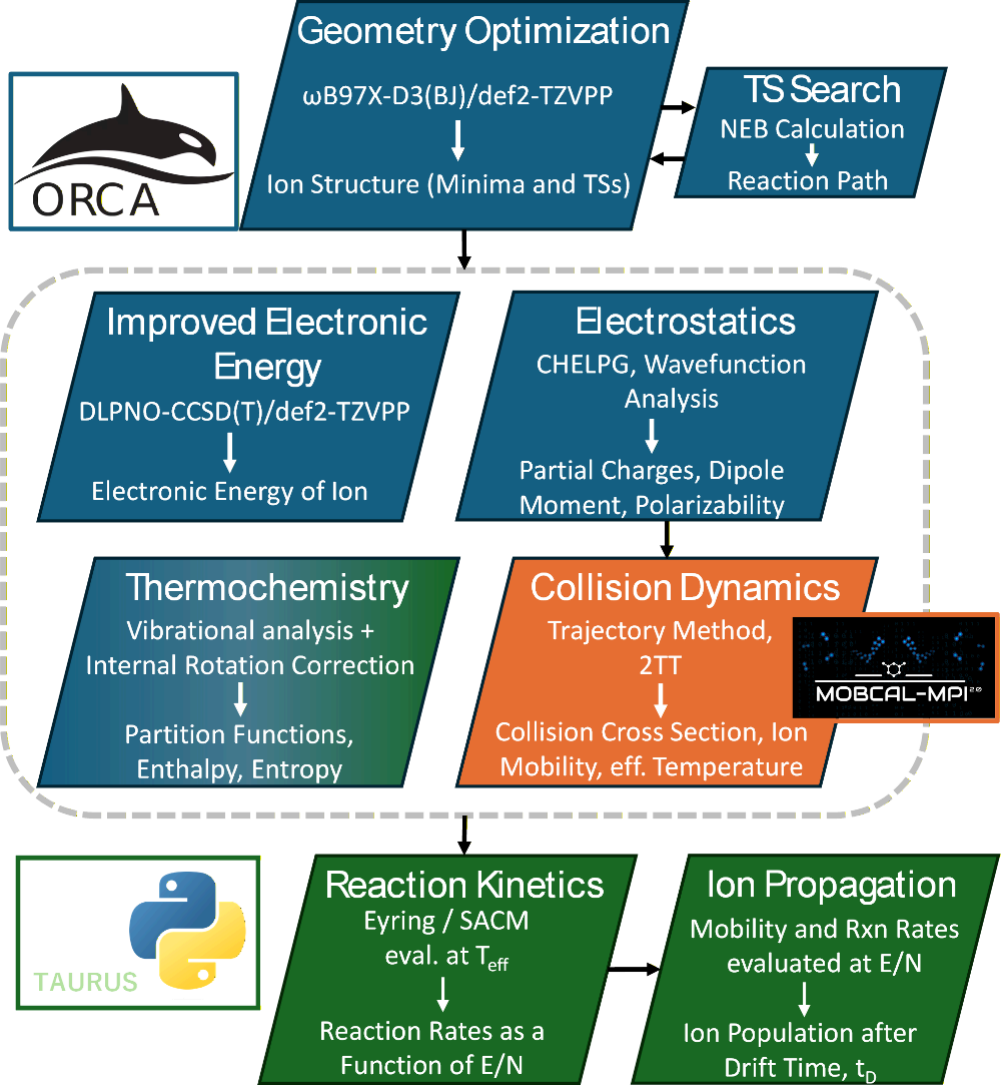
Flowchart of the computational modeling needed to predict ion populations, as measured experimentally. Green elements are home-written Python code (called TAURUS), while blue and orange elements use the ORCA and MobCal-MPI 2.0 programs, respectively.

### Experimental Methods

To verify the modeled ion dynamics, we conducted measurements on two different instruments. First, we utilize a HiKE-IMS system coupled to time-of-flight MS to investigate the field-dependent stability of the protonated alcohols and the identity of potential fragments. The HiKE-IMS-MS system used has been described elsewhere,^55^ a scheme of the setup can be found in the SI (Section S1.1), and operational parameters are defined in Table 1. Briefly, the HiKE-IMS is a drift tube IMS operating at ∼14 mbar and ambient temperature in which the reduced electric field strength can be varied between 20 and 120 Td, covering a broad range of ion activation. Analytes are introduced as vapors and get ionized in a reaction region by primary reactant ions formed by a corona discharge (mainly protonated water clusters, see Figure S6). The drift tube is connected to a time-of-flight (ToF) MS, allowing us to determine the *m*/*z* of the found parent and fragment ions as a function of the set reduced field strength in the drift region. All analytes were purchased from Sigma-Aldrich Germany with a reported purity ≥99% and used without further purification. Nitrogen gas was supplied using a nitrogen generator (NG5000A, Peak Scientific, U.K.) with an internal pressure swing absorber in series with an additional activated carbon filter (Supelcarb HC Hydrocarbon Trap, Supelco, U.S.A.) and a moisture trap (Molecular Sieve 5A Moisture Trap, Supelco, U.S.A.). Water and oxygen content in the provided nitrogen are <1 ppm_V_ and <0.5 ppm_V_, respectively. The relative humidity of the sample gas was set to 10% (referenced to 293.15 K and 1013.25 hPa) by mixing dry nitrogen with humidified nitrogen. While the drift gas was kept dry, previous studies on the HiKE-IMS-MS system indicate that the residual water volume fraction is likely around 70 ppm_V_.^55^ This value is used for the ion dynamics calculations shown later, as it influences the ion–water cluster association rates.

**Table 1:** Operating Parameters of the HiKE-IMS-MS

Parameter	HiKE-IMS-MS
Drift length	150.5 mm
Reaction region *E*/*N*	60 Td
Drift region *E*/*N*	20–120 Td
Pressure	14.3 mbar
Temperature	25–30 °C
Approx. H_2_O content	70 ppm_V_

In addition to the first system, we conduct measurements on an atmospheric-pressure tandem IMS system comprised of two drift tube IMS connected via a fragmenter region, as described elsewhere^31^ and schematically shown in the SI (Section S1.2). Here, analytes are again introduced as vapors and ionized through reactant ions produced by a 370 MBq ^63^Ni foil. Ions can then be mobility-selected in a first drift tube and then subjected to an electric field from a sinusoidal waveform applied to two wire grid sets separated by 0.5 mm, i.e., the fragmenter region. Fragment ions are then separated in a second drift tube, allowing us to study the structure of the formed fragments via their ion mobility coefficients. A uniform electric field is applied over the entire drift region, and the sinusoidal waveform is superimposed only in a small portion of the whole drift field. The peak-to-peak amplitude of the fragmenter waveform can be varied from 0 to 3000 V (0 to 166 Td peak reduced field strength at 80 °C and ambient pressure), enabling tuning of the ion activation. Important operational parameters are summarized in Table 2. Chemicals were purchased from Sigma-Aldrich Chemical Co. (Milwaukee, WI) with more than 97% purity. The drift gas of 318 mL/min air was supplied by an Aadco 737 pure air generator and further treatment through 5 Å molecular sieves. Using a Panametrics Air.IQ-1–1 moisture meter (Panametrics Corp., Shannon, Ireland), we measured the water content in the instrument to be ∼3 ppm_V_. Note that in the following we will report the drift times only in the second drift region, labeled “corrected drift time”, not the overall drift time through the whole instrument. For ion mobility calibration, we used 2,6-di-*tert*-butylpyridine, a stable, largely unclustered ion as a mobility coefficient standard.^56^

**Table 2:** Operating Parameters of the Tandem IMS Used

Parameter	Tandem-IMS
Drift length 1	47.1 mm
Fragmenter length	0.5 mm
Drift length 2	38.2 mm
Pressure	875 mbar
Temperature	78 °C
Drift field	456 V/cm
Ion gating window	400 μs
RF frequency	3.39 MHz
Approx. H_2_O content	3 ppm_V_

## Results and Discussion

### Fragmentation Dynamics of Secondary Alcohols

To start, we investigate the simpler case of secondary alcohols for later comparison to primary alcohols. Water loss of protonated secondary alcohols yields stable secondary carbocations (R^1^-RH^+^-R^2^) and thus can occur via simple bond cleavage of the R–O bond. The simplest secondary alcohol is isopropyl alcohol (IPA) and PTR-MS studies have shown water loss for high reduced field strengths.^24^ Loss of water, yielding the isopropyl carbocation, H_3_C–CH^+^–CH_3_, proceeds via simple bond cleavage, i.e., a loose TS, whereby the interaction potential is dominated by the ion-dipole interaction of the two fragments due to the dipole moment of H_2_O. From our modeling, we find a threshold energy of Δε_0_ = 101 kJ/mol. Here, Δε_0_ refers to the difference in the zero-point-energy-corrected electronic energies.

The fragmentation dynamics already become more complicated when turning to larger secondary alcohols due to the increased flexibility of their side chains. In particular, the fragmentation thresholds depend on the conformation of the resulting fragment. The most stable structure of the 2-butyl carbocation, Et–CH^+^–Me, has been extensively studied both experimentally and theoretically.^57–60^ Most of these studies suggest that secondary carbocations (2-butyl and larger) show a so-called protonated cyclopropane (PCP^+^) moiety, where the empty *p*-orbital at the carbocation strongly interacts with the C–H group in the β-position. Figure 2 shows the structure and relevant (localized) bonding orbitals for the 2-butyl carbocation as obtained by the ωB97X-D3­(BJ)/def2-TZVPP level of theory used here. The side chain of larger secondary carbocations can thus stabilize the charge by folding back on itself.

**Figure 2:**
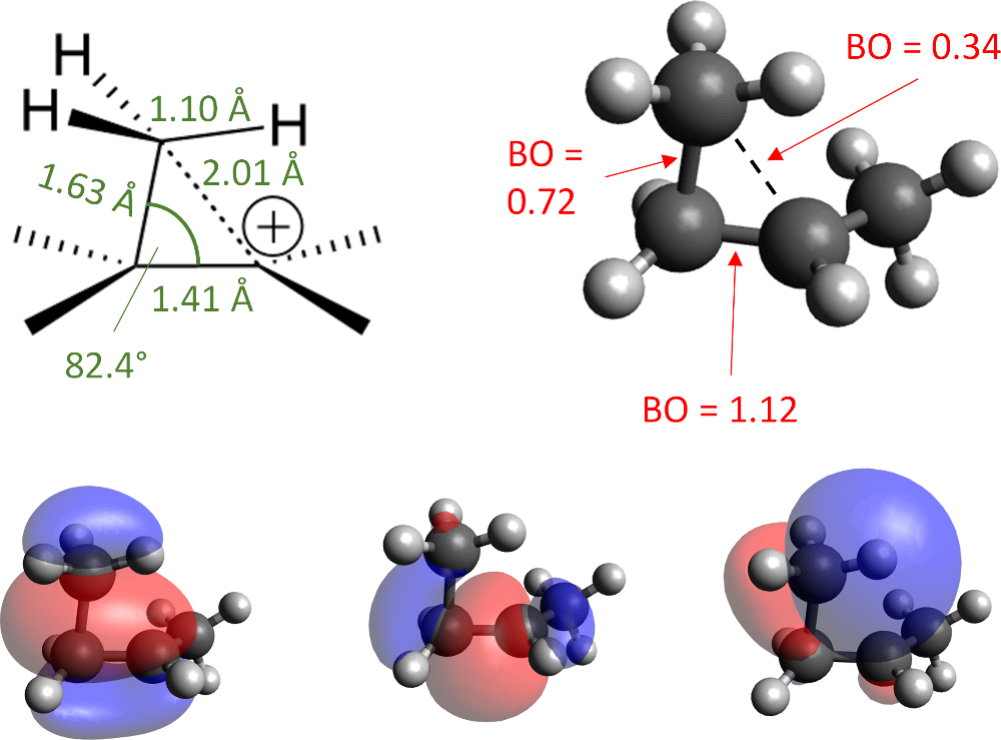
Structure and selected bonding orbitals of the 2-butyl carbocation. Orbitals are localized molecular orbitals (LMOs) and bond orders are Mayer bond orders.

This influences the fragmentation dynamics, as this charge stabilization can already occur during the fragmentation process. We found a pseudosubstitution for protonated 2-butanol, where the −OH_2_ group is cleaved as the PCP^+^ moiety forms, proceeding via a tight TS with a barrier height of only Δ*ε*_0_ = 57 kJ/mol. This first yields an electrostatically bound dissociative complex (DC), which then quickly dissociates to the separated products via a loose TS with a threshold energy of only Δ*ε*_0_ = 46 kJ/mol. The total bond dissociation energy amounts to Δ*ε*_0_ = 92 kJ/mol, significantly lower than that for IPA. The fragmentation mechanism and corresponding potential energy surfaces are shown in Figure 3A and B.

**Figure 3:**
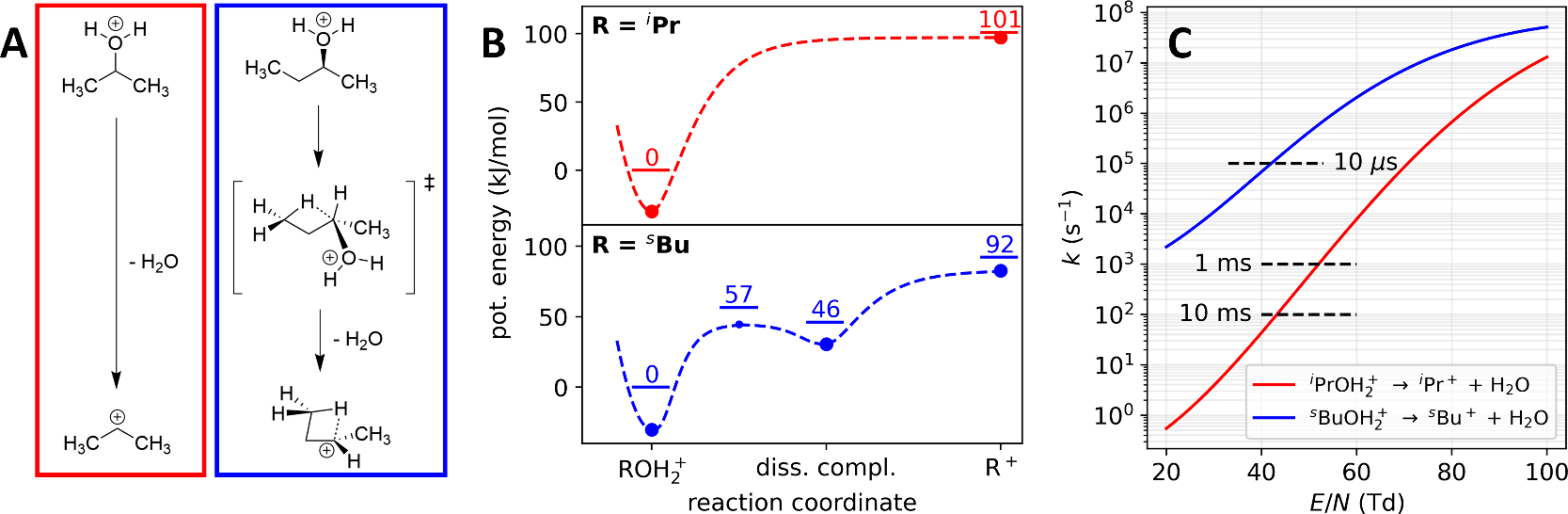
(A) Fragmentation mechanism, (B) potential energy surface with zero-point energy levels superimposed, and (C) corresponding fragmentation rates at 14.3 mbar and 300 K for protonated ^i^PrOH (red) and ^s^BuOH (blue).

Figure 3C compares the computed rates of fragmentation for protonated 2-propanol (bond cleavage) and protonated 2-butanol (pseudosubstitution followed by dissociation) as a function of the ion activation, represented by the reduced field strength, *E*/*N* (see Figure S3 for *T*_eff_ vs *E*/*N* plots). It becomes clear that favorable stabilization of the PCP^+^ moiety in protonated 2-butanol accelerates the fragmentation rate significantly. While protonated 2-propanol is predicted to be stable up to 50 Td for instrumental time scales shorter than 1 ms, protonated 2-butanol basically becomes instable for any significant ion heating.

For larger secondary alcohols, we expect similar energetics as for 2-butanol, since they can stabilize the charge via the PCP^+^ moiety as well. Indeed, the dissociation thresholds are very similar for protonated 2-butanol, 2-pentanol, 2-hexanol, and 2-heptanol (see below). The fragmentation rates, however, should be smaller the larger the alcohol when plotted vs E/N as their lower ion mobility yields lower effective temperatures (cf. eq 1), i.e., at a given field strength, protonated 2-pentanol would experience less ion activation than protonated 2-butanol and thus fragment slower.

### Fragmentation Dynamics of Primary Alcohols

The fragmentation of protonated primary alcohols seems less straightforward as a simple bond cleavage would result in an unstable primary carbocation.^25^ Plenty of rearrangement reactions of carbocations are known in the literature,^60,61^ and thus, we first hypothesized that primary carbocations would form through simple bond cleavage and subsequently rearrange to secondary carbocations. However, our DFT modeling showed that primary carbocations are not stable in the gas phase; i.e., they do not represent a local minimum on the potential energy surface. Instead, all geometry optimizations lead to the formation of secondary carbocations via hydride shifts. Indeed, if the fragmentation mechanism would initially pass through a primary carbocation, protonated ethanol should show fragmentation just as protonated *n*-propanol. Yet, this is not the case.^24^ Given the pseudosubstitution pathway found for protonated 2-butanol (cf. Figure 3A), the structure of the PCP^+^ moiety, and other reports on intramolecular substitutions of primary protonated hydroxyl groups,^19^ we investigated a pathway where a hydride shift substitutes the protonated hydroxyl group, forming a secondary carbocation in a concerted mechanism (see Figure 4A). This indeed would explain the lack of water loss observed from protonated ethanol, as no secondary carbocation can form.

**Figure 4:**
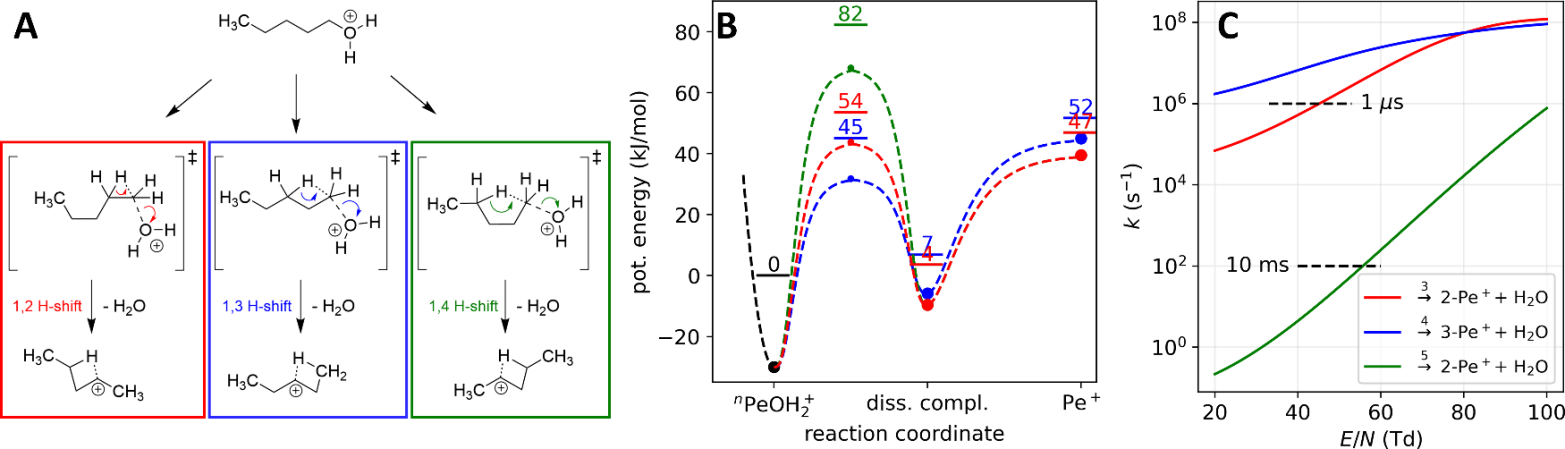
(A) Fragmentation mechanism, (B) potential energy surface with zero-point energy levels superimposed, and (C) corresponding fragmentation rates at 14.3 mbar and 300 K for protonated *n*-pentanol via 1,2-H shift (red), 1,3-H shift (blue), and 1,4-H shift (green). Note that the 1,2 and 1,4-H shifts yield the same product, namely, the 2-pentyl carbocation.

Our DFT modeling shows that these substitutions occur via a concerted TS, i.e., as the C–O distance increases, the C–H distance decreases, and the primary carbon center is inverted as in an S_N_2 reaction. See the SI for a movie. Depending on the chain length different hydride shifts are possible. The smallest hydride shift, a 1,2-H shift passing through a 3-membered TS, can already occur for protonated *n*-propanol. For protonated *n*-butanol and larger, a 1,3-H shift passing through a 4-membered TS can occur as well. The product of these substitutions is, again, a dissociative complex that then yields the separated products via a loose TS (see Figure 4B). To systematically investigate these different pathways, we calculated barrier heights for all possible hydride shifts for *n*-butanol, *n*-pentanol, *n*-hexanol, and *n*-heptanol. Their threshold energies and thermodynamic functions can be found in Table 3 and are exemplarily shown for *n*-pentanol in Figure 4B. The original work developing the used ωB97X-D3 functional reports a mean absolute error (MAE) of 8.7 kJ/mol for hydrogen transfer barrier heights (HTBH).^34^ We expect our uncertainties to be slightly better due to the improved dispersion correction and newer basis set.

**Table 3:** Characteristics of the Intramolecular Substitution Barriers for the Homologous Series of Protonated *n*-Alcohols, Including Transition State Ring Size (*R*), Threshold Energies (Δε_0_), Standard Enthalpy Change (Δ*H* ^‡,⊖^), and Standard Entropy Change (*ΔS* ^‡,⊖^)

	H-shift	*R*	Δ*ε*_0_ kJ/mol	Δ*H* ^‡,⊖^ kJ/mol	Δ*S* ^‡,⊖^ J/mol K
*n*-butanol	1,2	3	54.4	63.7	31.5
	1,3	4	49.8	58.5	37.2
*n*-pentanol	1,2	3	53.6	57.0	31.6
	1,3	4	45.1	48.8	36.4
	1,4	5	82.2	83.9	12.4
*n*-hexanol	1,2	3	53.0	56.5	32.0
	1,3	4	42.9	46.8	37.2
	1,4	5	79.1	80.7	11.7
	1,5	6	50.1	50.3	–2.1
*n*-heptanol	1,2	3	52.9	56.3	31.8
	1,3	4	42.1	45.9	37.1
	1,4	5	78.0	79.5	11.3
	1,5	6	47.8	47.9	–3.0
	1,6	7	62.7	62.5	–3.9

Comparing the same H-shift for different chain lengths shows little variation; i.e., the length of the chain has little influence on a given hydride shift. Comparing different hydride shifts for the same molecule, we can see that the enthalpy and entropy changes differ significantly, even given the estimated uncertainties. Specifically, the change in entropy generally decreases with the TS ring size, meaning that larger rings are entropically less favored. This aligns with chemical intuition, as larger ring-TSs require a large portion of the carbon chain to fold back onto the primary carbon, reducing conformational flexibility and thus the density of states. However, in contrast to chemical intuition, the enthalpy change does not show a clear trend with the ring size. Typically, one would expect that larger rings impose less geometrical stress and are thus more favorable; yet, this is not observed here. The 1,4-H shifts (5-membered rings) seem to be the least favorable, while the 1,3-H shift seems to be the most favorable across the analytes studied.

The 1,3-H shift passes through a 4-membered TS, which means that the most stable conformation of the carbocation, the PCP^+^ moiety, is already preformed in the TS. By Hammond’s postulate, it follows that the barrier height should be somewhat small, which is consistent with our observations. A deeper analysis shows that the bond orders and bonding orbitals in the 4-membered TS are very similar to the ones in the respective carbocation (see Figure S4), highlighting that this H-shift is specifically favored. Given the somewhat large uncertainties of the barrier heights, other H-shifts are likely also possible, especially at larger ion energies. In particular, the 1,5-H shift passing through a 6-membered TS seems low in energy, likely due to minimal geometric strain (as is common for 6-membered rings). This leaves the impression that all even-numbered ring TSs have lower barriers than odd-numbered ring TSs. However, this might just be due to the favorable configurations in the 4-ring and 6-ring TSs and not a general trend.

Computing the field-dependent rate coefficients for the different H-shifts, as shown exemplarily for *n*-pentanol in Figure 4C, we see the threshold energies being reflected in the intercepts and the entropy changes being reflected in the slopes of the curves. In particular, it becomes clear that the pathway via the 4-membered TS is dominant over the other pathways. Only at very high reduced field strengths does the 3-membered ring TS pathway start to compete with the 4-membered ring TS. Importantly, our simulations predict that on typical instrumental time scales of 1–10 ms, the protonated primary alcohols are not stable even at mild ion activation conditions. For example, *n*-pentanol experiences a fragmentation lifetime of only 1 μs at 20 Td (300 K and 14.3 mbar), suggesting that after protonation, primary alcohols fragment very quickly. While quick fragmentation of protonated hydroxyl groups is well-known, these results are still surprising. Moreover, in contrast to solution phase chemistry, where secondary alcohols usually show higher reactivity than primary alcohols, we find that in the gas phase primary protonated alcohols fragment more easily.

### Influence of Background Moisture

In the early ion transfer stages or in ambient pressure IMS, a significant amount of background moisture is still present in the gas phase. This is important as, just as in the solution phase, water can stabilize charges through electrostatic interactions. Indeed, ion–water (or more generally ion–solvent) clusters are readily formed under typical conditions here considered.^32,62,63^ Thus, to draw a complete picture of the fragmentation dynamics of protonated alcohols, these interactions must be considered, as well.

To this end, we computed the structure, energetics, and ion mobilities of protonated alcohol–water clusters up to *n* = 2 water ligands, i.e., [R–OH_2_^+^ + *n*(H_2_O)] with *n* ≤ 2. These form a reaction network of successive association and dissociation of neutral water molecules onto the ion, as shown in Scheme 1 for 2-propanol. Note that the indicated equilibria are not necessarily reached. We observe that this is only the case at low *E*/*N*, where ion–water clusters are relatively stable and enough reaction events occur during the drift time.

**Scheme 1:**
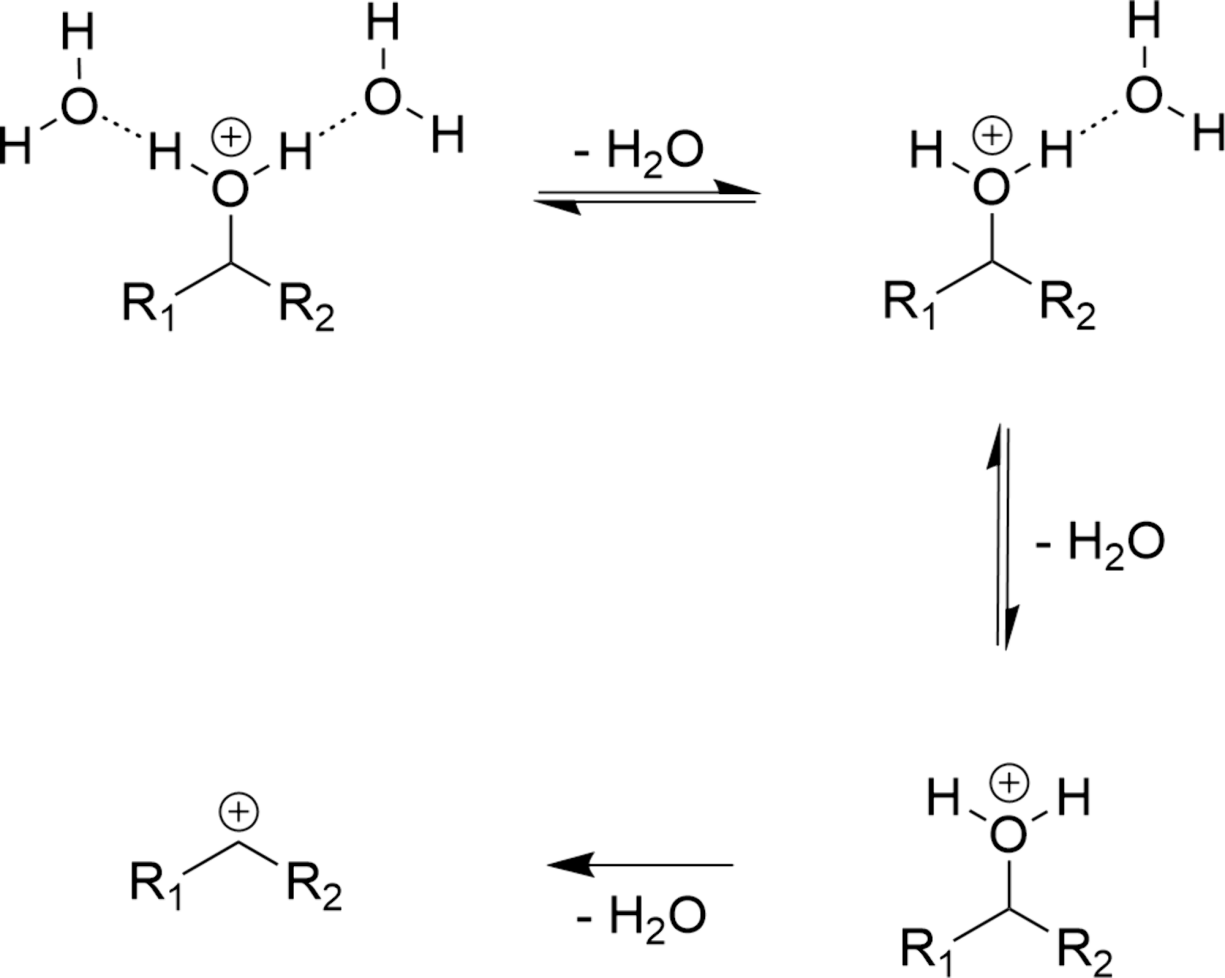
Reactions Involving Ion-Water Clusters Considered for the Simulations^a^

a Exemplarily shown for a secondary alcohol.

Considering first the binding energies of the different water loss steps, as shown in Figure 5, we find that the binding energies of the *n* = 2 clusters lie around 75 kJ/mol for the secondary alcohols and around 80 kJ/mol for the primary alcohols. This slight difference is more pronounced for the binding energies of the *n* = 1 clusters, where we find around 87 kJ/mol for the secondary alcohols but a strong concentration of 101 kJ/mol for the primary alcohols. This reflects the fact that the charge is less stabilized for the primary alcohols, thus needing a stronger solvation contribution. Ref 34 reports a MAE for noncovalent interactions of only 1.7 kJ/mol, giving us high confidence in the significance of these differences.

**Figure 5:**
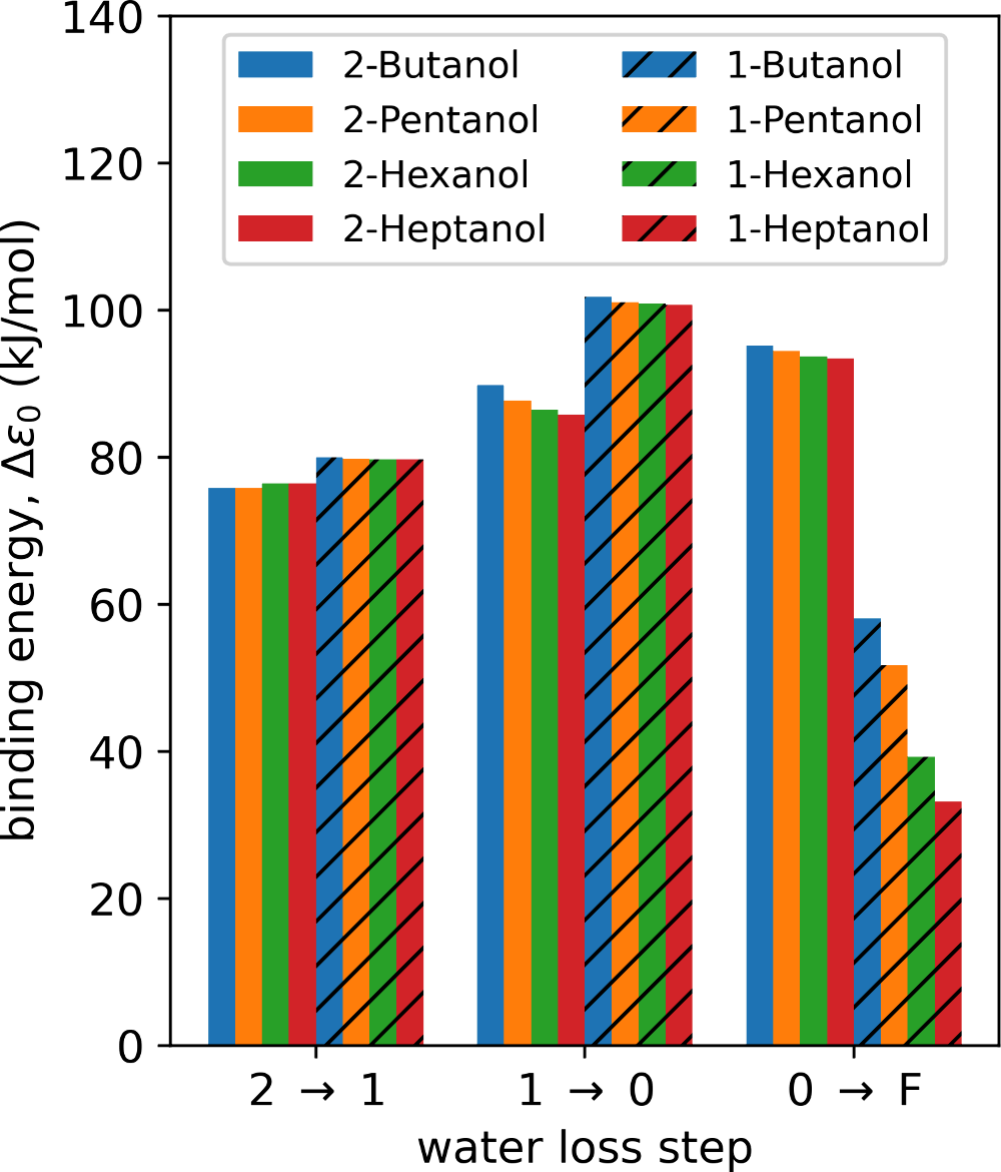
Binding energies of the different water loss steps of the secondary and primary alcohols. “2 → 1” corresponds to [R–OH_2_^+^ + 2­(H_2_O)] → [R–OH_2_^+^ + H_2_O] + H_2_O, “1 → 0” corresponds to [R–OH_2_^+^ + H_2_O] → R–OH_2_^+^ + H_2_O, and “0 → F” corresponds to R–OH_2_^+^ → R^+^ + H_2_O.

Comparing the binding energies of the ion–water clusters with the fragmentation energies of the protonated alcohols (taking the full energy difference between protonated ion and separated products), we find for the secondary alcohols that the fragmentation energy is only slightly higher than the cluster binding energy (94 kJ/mol vs 87 kJ/mol), and strikingly, for the primary alcohols, the fragmentation energy is actually less than the cluster binding energy (58–33 kJ/mol vs 101 kJ/mol). This means that while the bare protonated alcohols might readily fragment under mild ion activation conditions, the alcohol–water clusters are somewhat stable and might prevent fragmentation. Citing again ref 34, the MAE reported for the proton affinity (as a metric for general thermochemistry) is 4.6 kJ/mol, supporting the significance of these energies.

### Experimental Validation

For experimental validation of the found fragmentation dynamics, we first measured the ion population distributions (portion of parent and fragment ion as identified by their *m*/*z*) of 2-propanol and 1-butanol using HiKE-IMS-MS between 20 and 100 Td. Figure 6 shows the raw mass spectra of 1-butanol over the measured *E*/*N* range, indicating different ion species. Specifically, we observe protonated water clusters (reactant ions) at *m*/*z* 55 and 73, as well as protonated 1-butanol clustered with one (*m*/*z* 93) or two (*m*/*z* 111) water, and its water-loss fragment (*m*/*z* 57). The bare ion (*m*/*z* 75) is not observed at any reduced field strength.

**Figure 6:**
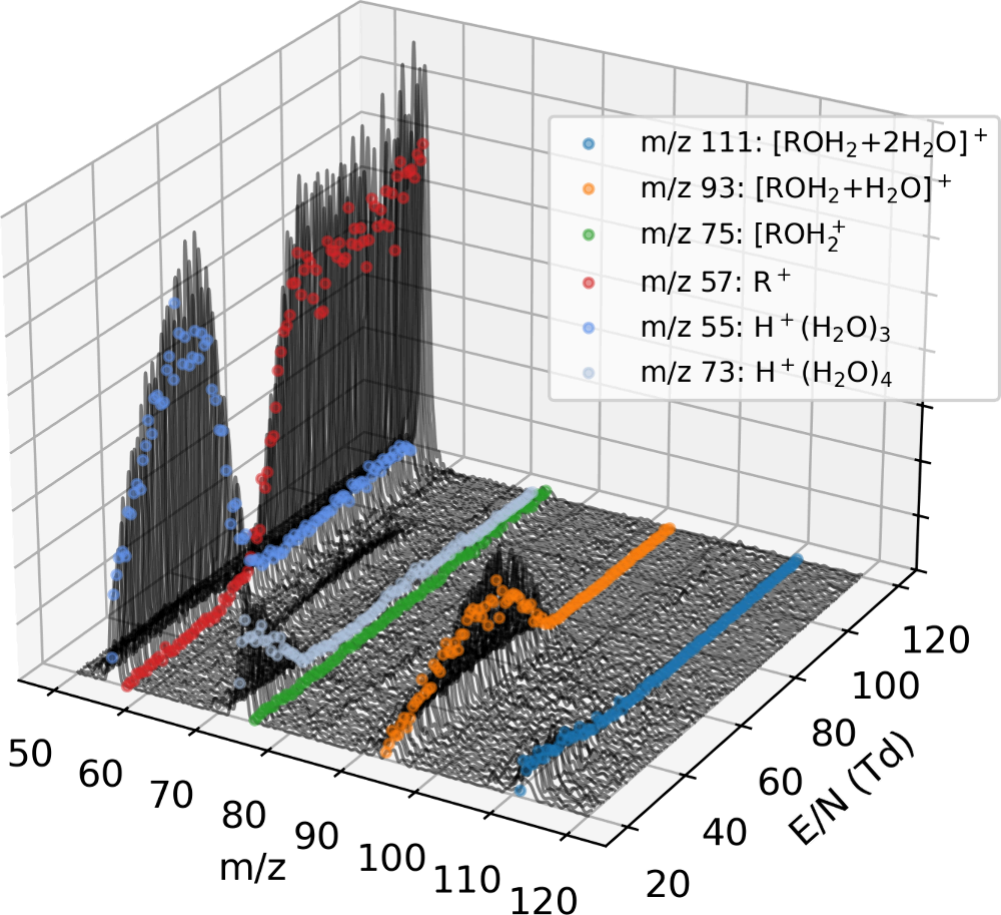
Waterfall plot of the recorded mass spectra of 1-butanol over a range of reduced field strengths, *E*/*N*. Colored traces indicate the ion counts at specific *m*/*z* values.

To further process the data, we fit the peak areas around the indicated masses for all mass spectra recorded and averaged them over three independent measurements. This yields, after normalization, the ion population distribution at the end of the drift tube. As we obtain the field-dependent reaction rates using our modeling, we can directly compute the measured ion population distribution by equating the total reaction time with the expected drift time at a given *E*/*N*.^3^ Figure 7 shows this comparison for 2-propanol and 1-butanol with respective mass spectra shown in Figure S7 and S8. Note that the colored traces shown in Figure 6 are peak amplitudes, whereas Figure 7 shows peak areas normalized for the ion species considered. At low ion activation, the data shows significant ion–water clustering ([ROH_2_ + *n*(H_2_O)]^+^) due to the background moisture present in the HiKE-IMS-MS of around 70 ppm_V_.^55^ Increasing the ion temperatures leads to a decrease in the average cluster size. This behavior is well reproduced by the simulations (also assuming 70 ppm_V_ of background moisture), indicating that the modeled ion–water binding strengths are well captured.

**Figure 7:**
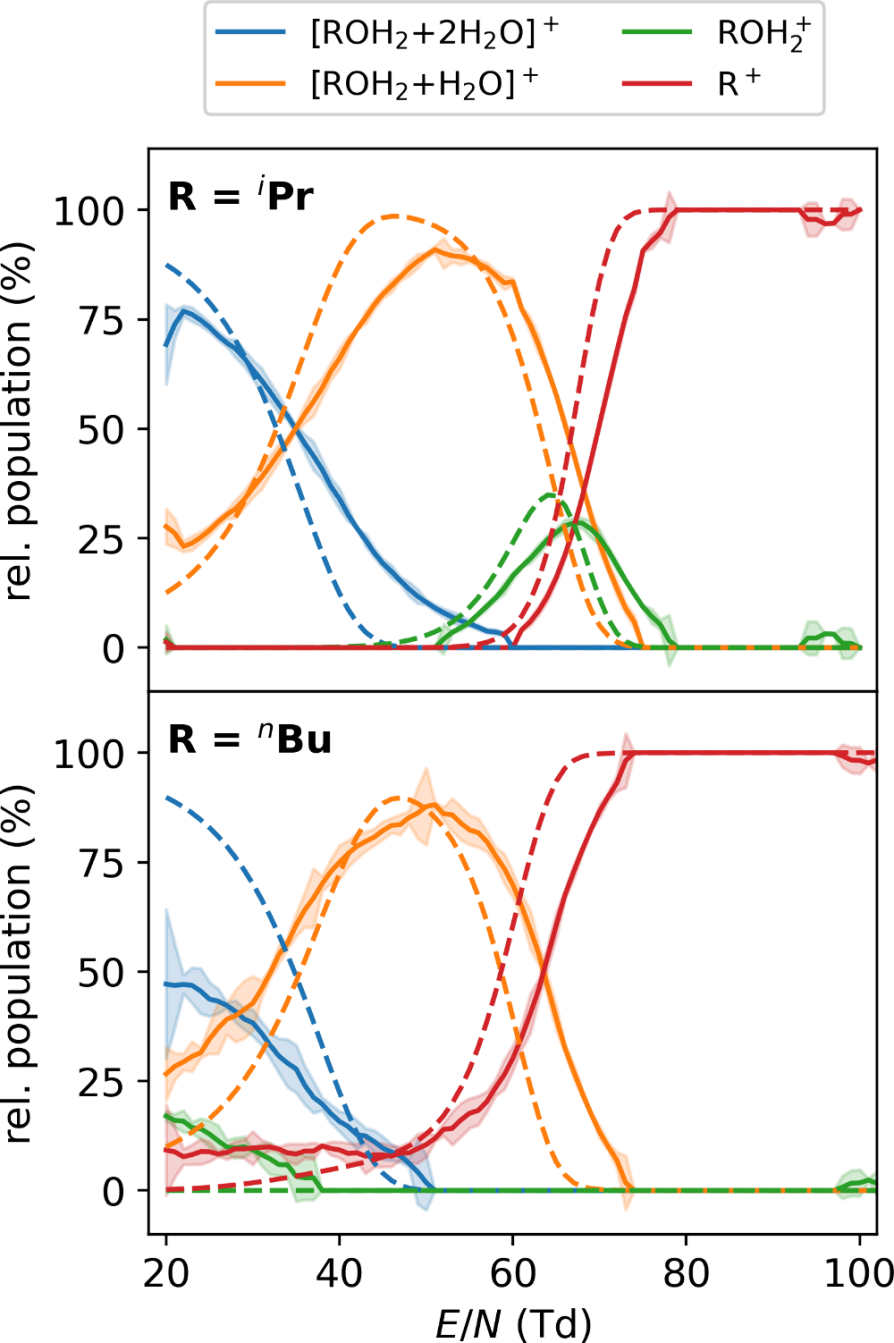
Measured (solid lines with error bars) and computed (dashed lines) ion populations of protonated 2-propanol (^*i*^PrOH) and 1-butanol (^*n*^BuOH) in the HiKE-IMS-MS over a reduced field strength range of 20–100 Td in the drift region.

Around 60 Td, the ion–water clusters for protonated 2-propanol become unstable and the bare ion is observed (and predicted by our model). This is in line with the computed rate coefficients (cf. Figure 3C): At 60 Td, the lifetime of protonated 2-propanol is around 130 μs and is thus comparable to the drift time of 400 μs at this *E*/*N*, i.e., the fragmentation is still slow enough compared to the experimental time scales that the bare ion can be observed. As the ions enter HiKE-IMS as ion–water clusters, the initial dissociation of these clusters further prolongs the effective lifetime of the bare ion. Increasing the ion activation further, the fragmentation lifetimes of protonated 2-propanol decrease enough that the carbocation, ^*i*^Pr^+^, is observed.

In contrast, the fragmentation lifetimes of primary protonated alcohols are much shorter due to the small binding energies (cf. Figure 5) and the small barrier of the found intramolecular S_N_2 reaction (cf. Figure 4). Again taking 60 Td as an example, the lifetime of bare protonated 2-butanol is only around 0.1 μs according to our computed rate coefficients. Consequently, as soon as the ion–water clusters dissociates, the bare ion fragments quickly and is not observed in the experiment (or simulation) (see Figure 7). Even at only 20 Td, the computed fragmentation lifetime is a mere 5 μs and thus well below the experimental drift time. Thus, only through protective clustering with background water can we detect the unfragmented ion. This effect was also observed for protonated 1-octanol in low-field IMS-MS coupling: Activation of the hydrated analyte in the transfer region yielded directly the R^+^ fragment and no bare ion was observed.^26^ More data can be found in the SI, namely HiKE-IMS-MS measurements and modeling for protonated 1-pentanol (Figure S9) and 2-hexanol (Figure S10), showing consistent results.

The good agreement between the experimental and simulated ion population distributions is strong evidence that the computed binding energies are accurate. In particular, the lack of the bare protonated 1-butanol and 1-pentanol in the experiment supports the finding that a secondary carbocation is formed instead of a primary one, which would be much higher in energy, thus increasing the binding energy (and thus stability of the bare ion). Moreover, the experimental data also support the fact that the rearrangement reaction to form these secondary carbocations cannot have a barrier much higher than the fragmentation threshold. If this were the case, the bare ions would be kinetically stabilized by this high barrier, which is not observed. This is in line with the found intramolecular S_N_2 reaction, where our computed barriers (at least the ones of the 1,3-H shifts) are below or around the fragmentation threshold (cf. Figure 4 and Figure S5).

To further support the found intramolecular S_N_2 reaction, we conducted experiments using a tandem-IMS system with a fragmenter region on both protonated 1-butanol and 2-butanol to check whether, as predicted by our modeling, they yield the same secondary butyl carbocation. Figure 8 shows the experimental corrected arrival time distributions (ATD) of protonated 1-butanol and 2-butanol, respectively, over a range of ion activation RF_pp_ voltages. First, we can see that the arrival time of the precursor ions (protonated 1-butanol and 2-butanol) have good reproducibility and that the fragmenter waveform amplitude has no significant effect on the overall drift time. Protonated 1-butanol and 2-butanol are clearly separable in this setup with reduced mobilities of 1.79 cm^2^/(V s) and 1.85 cm^2^/(V s), respectively, albeit not baseline separated. While these values do vary with temperature and water content (due to the shifting effect of ion–water clusters on the ion mobility^64^), these values are in reasonable agreement with literature reports.^28^

**Figure 8:**
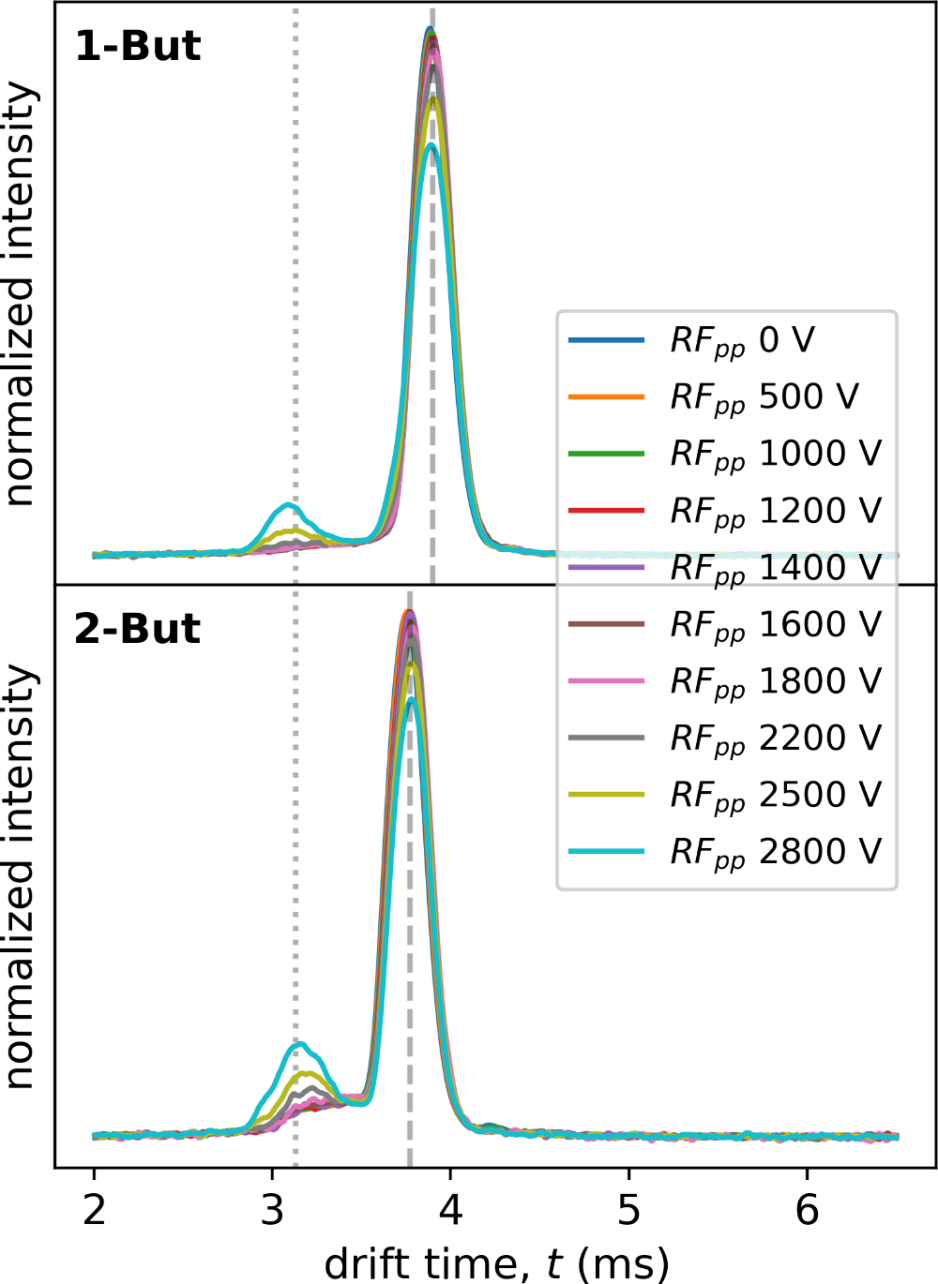
Tandem IMS arrival time distributions for protonated 1-butanol and 2-butanol over a range of ion activation RF_pp_ voltages in the fragmenter region.

Both spectra show a high-mobility fragment peak appearing as the RF_pp_ voltages are increased. While the mobility of this peak (∼2.2 cm^2^/(V s)) shows a greater variability due to the low intensity (see Figure S11), the data strongly suggests that this is the same fragment ion produced. In the literature, a number of different mobility values for (sometimes multiple) fragment peaks for protonated 1-butanol and 2-butanol are reported, ranging form 2.02 – 2.27 cm^2^/(V s).^10,28,31,65^ Shokri et al.^28^ also found the same fragment mobility (within experimental error) for both protonated 1-butanol and 2-butanol, consistent with our findings. In the future, we aim to couple our tandem IMS system to an MS for an unambiguous assignment of fragment identity. Still, we are confident, given the combination of modeling and experimental data, that the observed fragment is the same secondary butyl carbocation for both protonated 1-butanol and 2-butanol.

Interestingly, the fragmentation of the two protonated butanol isomers occurs only at quite high field strengths despite the elevated temperatures. Further protonated 2-butanol seems to show stronger fragmentation than 1-butanol, even showing a slightly elevated baseline between precursor and fragment, hinting at a constant thermal decomposition. This seems to be in contrast with the faster fragmentation rates of the primary protonated alcohols (cf. Figure 4 and Figure 3) and the short lifetimes of both. However, we measured a moisture level of 3 ppm_V_ (at atmospheric pressure) in the tandem IMS, which yields a high cluster association reaction rate (*k*_ass_[H_2_O] ≈ 1.2 × 10^5^ s^–1^, modeled value). This means that the lifetime of the unhydrated ion with respect to fragmentation becomes longer than the lifetime with respect to hydration, yielding relatively stable hydrated ions. Indeed, from our modeling we find that for the conditions of the tandem IMS system, the average number of water molecules attached to the protonated alcohols should be 1.7 for protonated 1-butanol and 1.2 for protonated 2-butanol. These ion–water clusters and their constant reformation due to collisions with neutral background water effectively protects the protonated alcohols from fragmenting up until very high RF_pp_ voltages. Moreover, consistent with the ion–water binding energies (cf. Figure 5) protonated 1-butanol is shielded more effectively due to stronger clustering, explaining the weaker amount of fragmentation observed in the tandem IMS data.

## Conclusions

Field-induced fragmentation of labile ions in medium to high pressure regions of mass spectrometers or ion mobility spectrometers can lead to misinterpretation of spectra but can also be used to increase specificity. In either case, understanding the dynamics, i.e., the molecular mechanisms and reaction rates, is crucial.

In this study, we focused on the dynamics of field-induced fragmentation of primary and secondary protonated alcohols as models for hydroxyl containing analytes. We found that protonation significantly weakens the C–O bonds, leading to ready loss of H_2_O. In other words, the reaction rates for water loss can be quite high, rendering protonated alcohols to be quite unstable species.

Mechanistically, we found that the protonated cyclopropane (PCP^+^) moiety plays an important role in the carbocation chemistry involved. For example, we found all formed carbocations to show this moiety, stabilizing their charge. Moreover, the fragmentation of primary protonated alcohols even undergoes an intramolecular S_N_2 reaction involving PCP^+^, leading directly to a secondary carbocation instead of an unstable primary carbocation. These charge stabilizations also affect the reaction rates by lowering fragmentation barriers/thresholds.

Background water (or other solvents) can significantly stabilize labile ions through the formation of ion–solvent clusters that first need to be dissociated before the ion can fragment. Their binding strength, influenced by charge localization/delocalization, plays a crucial role.

Overall, protonated hydroxyl groups are very easily cleaved through field-induced ion heating when they are not protected by a microsolvation shell. The ion geometric structure and charge localization/delocalization can significantly alter the fragmentation rates through their influence on the fragmentation barriers/thresholds, highlighting the importance of mechanistically understanding fragmentation processes for an analyte in question.

## Supplementary Material







## Data Availability

Structures of all calculated ions (including transition states) and neutrals are available through the ioChem-BD database^66,67^ under DOI: 10.19061/iochem-bd-6-594.

## Notes

More details on the experimental setups, the carbocation chemistry, more experimental data, as well as the Monte Carlo framework (PDF)
Ion mobility data (incl. *K*_0_, *T*_eff_, and 3rd order correction factors) (XLSX)
Minimum-energy pathway for the intramolecular S_N_2 reaction of protonated 1-butanol (GIF)
