# Supplementary material for: Exploring Field-Induced Fragmentation of Protonated Alcohols: Mechanistic Insights and Stabilizing Ion–Solvent Clusters
Source: J Am Soc Mass Spectrom. 2025 Dec 15;37(1):310–20. doi: 10.1021/jasms.5c00348 (PMC12784389; doi:10.1021/jasms.5c00348)
Supplement: Supplementary file 1 [file js5c00348_si_001.pdf]

Supporting Information

for

Exploring Field-Induced Fragmentation of Protonated Alcohols: Mechanistic Insights and Stabilizing Ion-Solvent Clusters

Philip Timmermann[a], Anjita G C Paudel[b], Gary Eiceman[b], Stefan Zimmermann[a], Alexander Haack[a]*

[a] Department of Sensors and Measurement Technology, Institute of Electrical Engineering and Measurement Technology, Leibniz University Hannover, 30167 Hannover, Germany

[b] Department of Chemistry and Biochemistry, New Mexico State University, Las Cruces, NM, 88003, USA

* Correspondence to: haack@geml.uni-hannover.de

Table of Contents

S1: Experimental Setups 3

S1.1 HiKE-IMS-MS Setup 3

S1.2 Tandem IMS Setup 4

S2: Properties of the Protonated Alcohols 5

S2.1: Effective Temperatures 5

S2.2: Protonated Cyclopropane 6

S2.3: PES of primary alcohols 7

S3: Additional HiKE-IMS-MS Data 8

S3.1: Blank Mass Spectra 8

S3.2: 2-Propanol 9

S3.3: 1-Butanol 9

S3.4: 1-Pentanol 10

S3.5: 2-Hexanol 10

S4: Peak Position Analysis for 1- and 2-Butanol 11

S5: Modeling of the HiKE-IMS-MS data 11

References 13

S1: Experimental Setups

S1.1 HiKE-IMS-MS Setup

The HiKE-IMS-MS system used here has been described previously.[1,2] Briefly, the system is comprised of a reaction region, a drift region, a transfer stage and the time-of-flight mass spectrometer, see **Figure S1**. The pressure in the reaction and drift regions are around 14 mbar, the temperature is ambient. A corona needle produces primary ions that quickly undergo transformation to the main reactant ions, namely $H^+(H_2O)_n$ and $NO^+(H_2O)_m$. The ions are introduced into the drift region via a tristate ion shutter.[3] The reduced field strength in the reaction region and drift region can be varied between 20 and 120 Td.

To measure the field-dependent ion populations, we kept the reduced field strength in the reaction region constant (60 Td), and varied the reduced field strength in the drift region alone. All ion gates were kept open such that a constant flow of ions reached the MS.

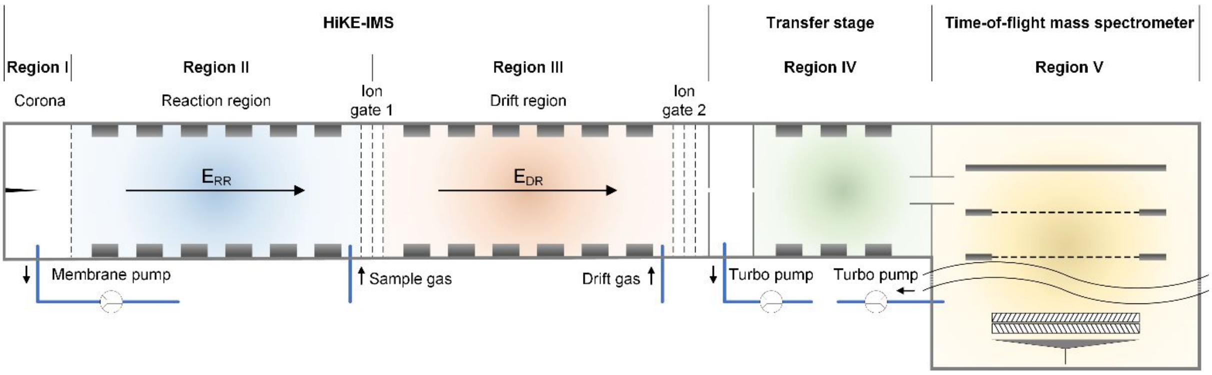

Figure S1: Scheme of the used HiKE-IMS-MS setup.

S1.2 Tandem IMS Setup

A schematic of the tandem IMS drift tube is shown in **Figure S2**. It is comprised of a reaction region, a first drift region, a fragmenter region, and a second drift region. The ion source is a planar stainless-steel disk with a 3 mm diameter center hole and is plated with 370 MBq of radioactive ^{63}Ni. Protonated monomer ions can be mobility-isolated using the first drift tube via ion shutter 2 by using a delay between ion shutter 2 and ion shutter 1 corresponding to a drift time appropriate of a specific ion peak. The pulse widths were 400 µs for both ion shutters. The mobility-isolated protonated monomer ions are then subjected to a tunable, high-amplitude sinusoidal waveform in the fragmenter region to facilitate ion activation. The fragmenter region is comprised of two metal etched grids 0.5 mm apart, placed at 3 mm from ion shutter 2. The formed fragments are then mobility separated in the second drift region for analysis. Ions are detected on a Faraday plate and the signal is amplified with a gain of ~10^{10} V/A. The signal is digitized using a ADC card, National Instruments model 6281. The electric field strength was uniform through the tandem drift tube at 456 V/cm.

The tandem IMS is operated at ambient pressure (660 torr) and at elevated temperatures, which is controlled through a resistive wire heater on the protective shell surrounding of the drift tube.

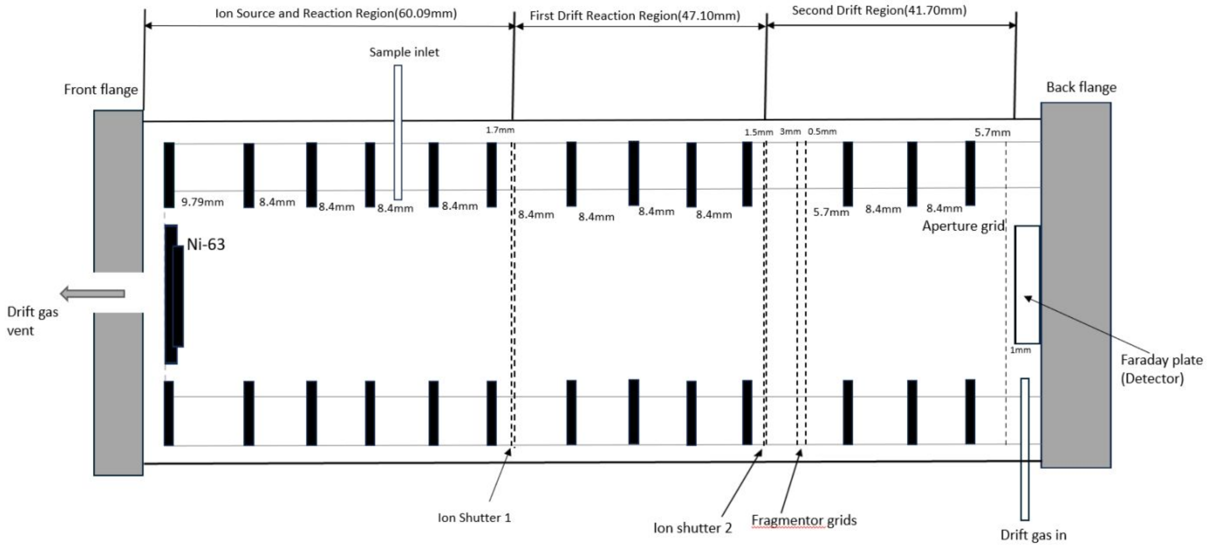

Figure S2: Scheme of the used tandem IMS setup with fragmenter region.

S2: Properties of the Protonated Alcohols

S2.1: Mobility Data

The description of the ion mobility and effective temperatures used here are:

$$[K_0]_3 = \frac{3}{16}\left(\frac{2\pi}{\mu k_B\left[T_{eff}\right]_3}\right)^{1/2} \frac{ze}{N_0\Omega\left(\left[T_{eff}\right]_3\right)}\left(1+[\alpha_{2TT}]_3\right)(S1)$$

$$\left[T_{eff}\right]_3 = T_{bath} + \frac{M}{3k_B}\left([K_0]_3 N_0 \frac{E}{N}\right)^2\left(1+[\beta_{2TT}]_3\right)(S2)$$

Whereby the brackets $[\cdots]_3$ indicate that we're using 3rd order two-temperature theory (2TT). Using the data obtained through MobCal-MPI 2.0, i.e., the CCSs, Ω,[4] we can compute T_{eff}, K_0, α_{2TT}, and β_{2TT} as a function of reduced field strength. **Figure S3** shows these data for all protonated alcohols investigated in this study. As can be seen, smaller ions with a higher mobility experience higher effective temperature as larger ions. Moreover, at high reduced field strengths, effective temperatures above 800 K are easily reached by small ions. The correction terms α_{2TT} and β_{2TT}, which are both zero at zero field strength and arise through the 3rd order approximation, usually stay well below 0.02 (absolute).

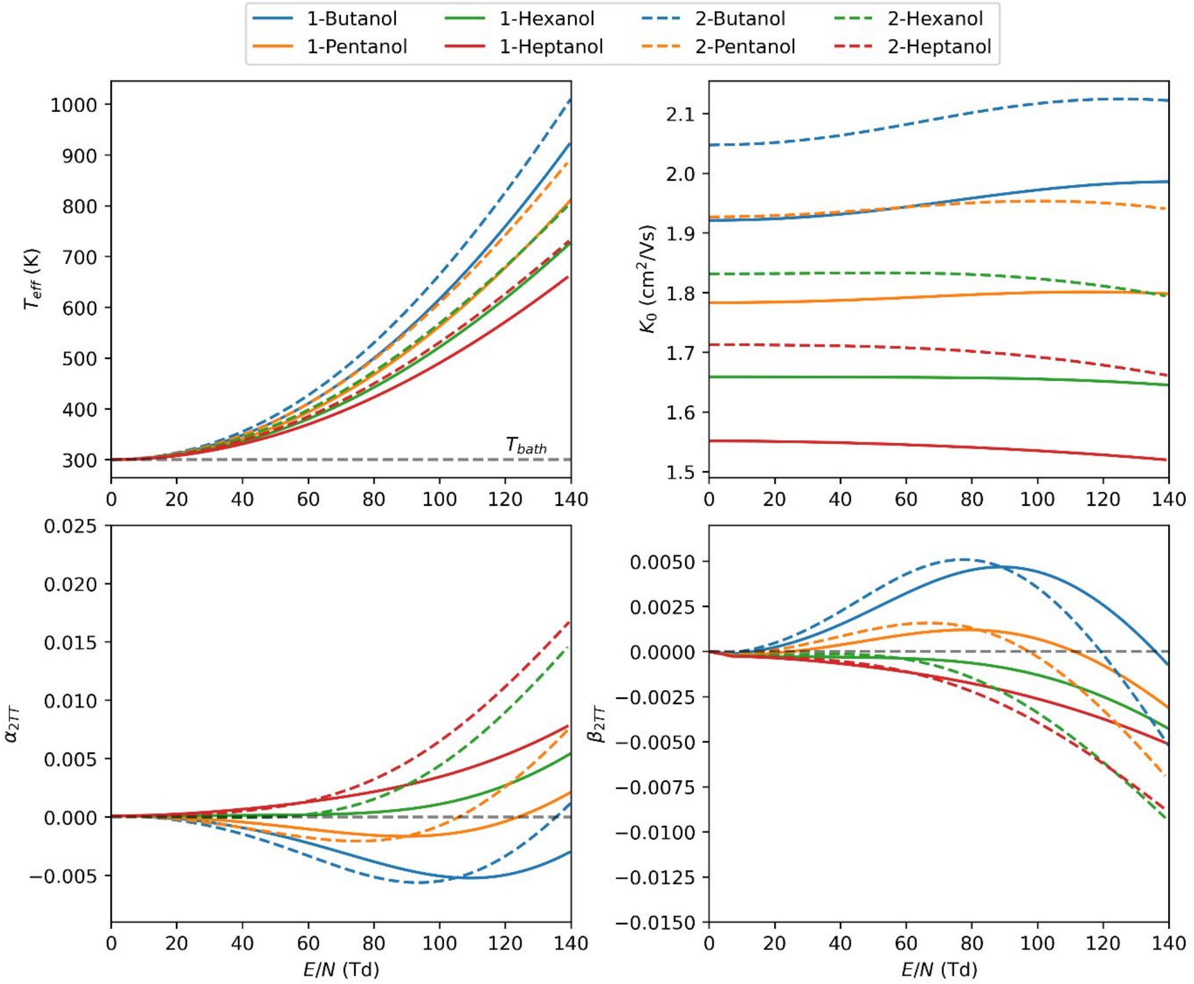

Figure S3: Effective temperatures of the protonated alcohols as computed through 3rd order 2TT using the data obtained from MobCal-MPI 2.0.

S2.2: Protonated Cyclopropane

To understand why out of all possible intramolecular S_N2 reactions, the 4-membered transition state is the most favorable, we investigated the electronic structure of the transition state and compared it to the one of the final fragment. This comparison is shown in **Figure S4**. Comparing the geometry, Mayer bond orders and corresponding localized molecular orbitals, one can see a great similarity, meaning that the transition state already closely resembles the final reaction product. By Hammond's postulate we can thus expect a small barrier. It is the stabilization of the charge through the formation of the PCP^+ moiety that effectively overcomes the geometric strain of a 4-membered TS.

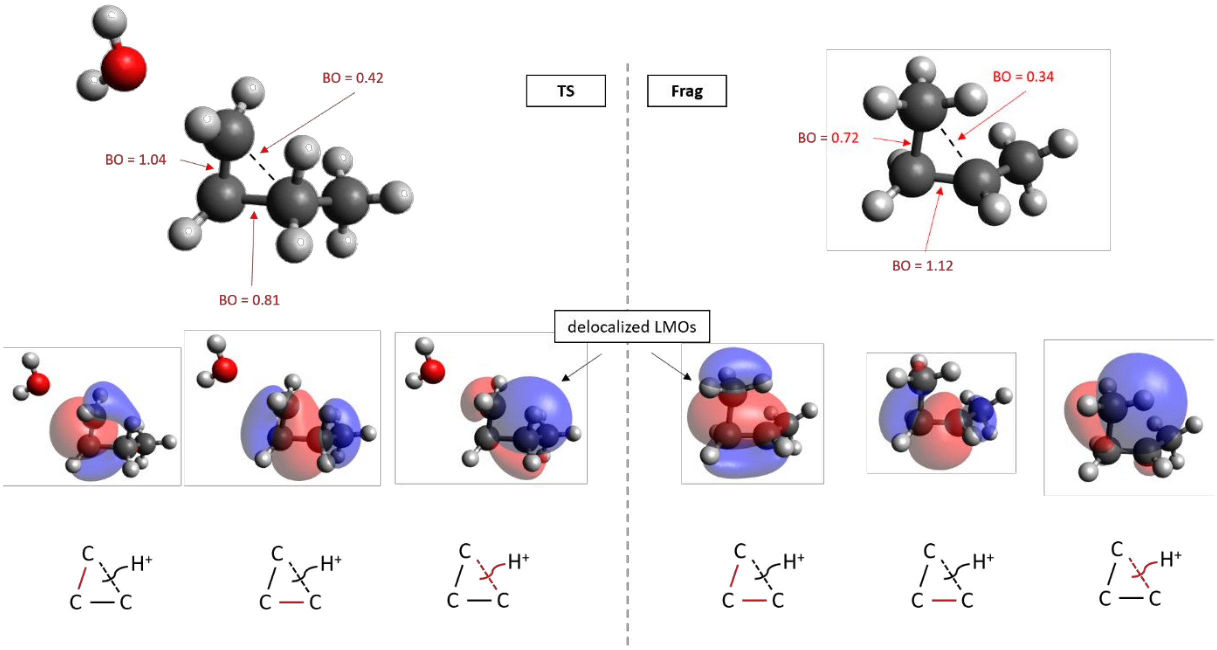

Figure S4: Comparison of the electronic structure of the 4-membered intramolecular S_N2 transition state (left) and the final 2-butyl carbocation fragment (right). Shown are the geometric structures and Mayer bond orders, the relevant localized molecular orbitals, and the bonds they most contribute to.

S2.3: PES of primary alcohols

We find that the intramolecular SN2 reaction can most easily proceed via a 4-membered transition state. The corresponding barriers are found to be below or just above the final threshold energies for dissociation as can be seen in **Figure S5**.

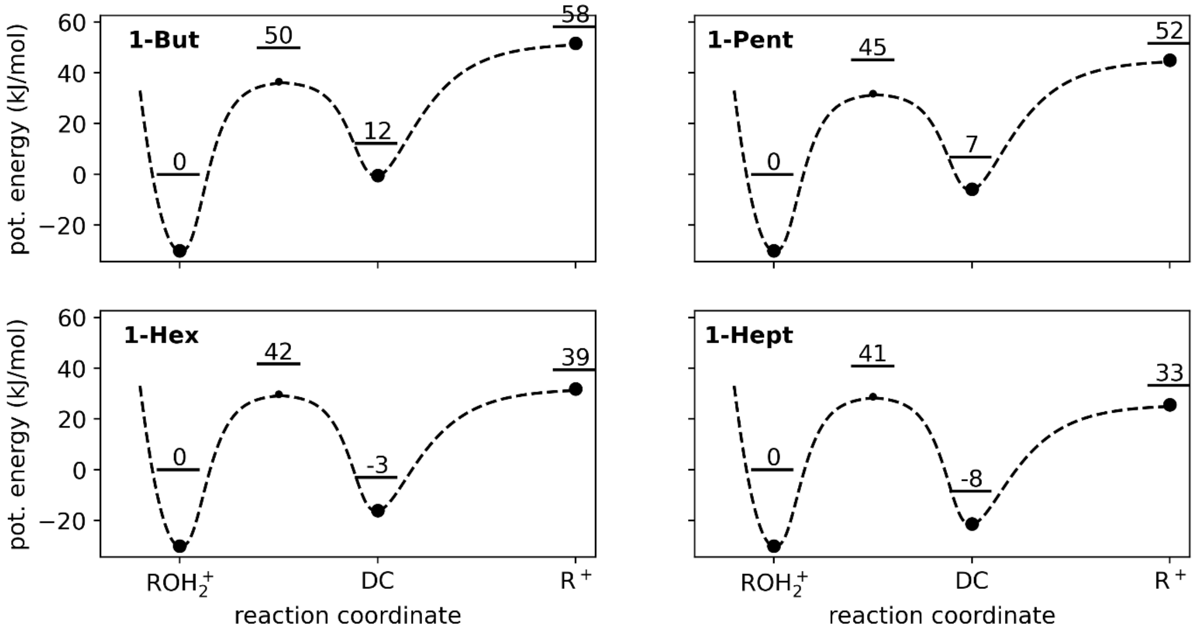

Figure S5: Potential energy surface with zero-point energy levels superimposed of the four primary alcohols studied. All transition states correspond to the 4-membered TS.

S3: Additional HiKE-IMS-MS Data

S3.1: Blank Mass Spectra

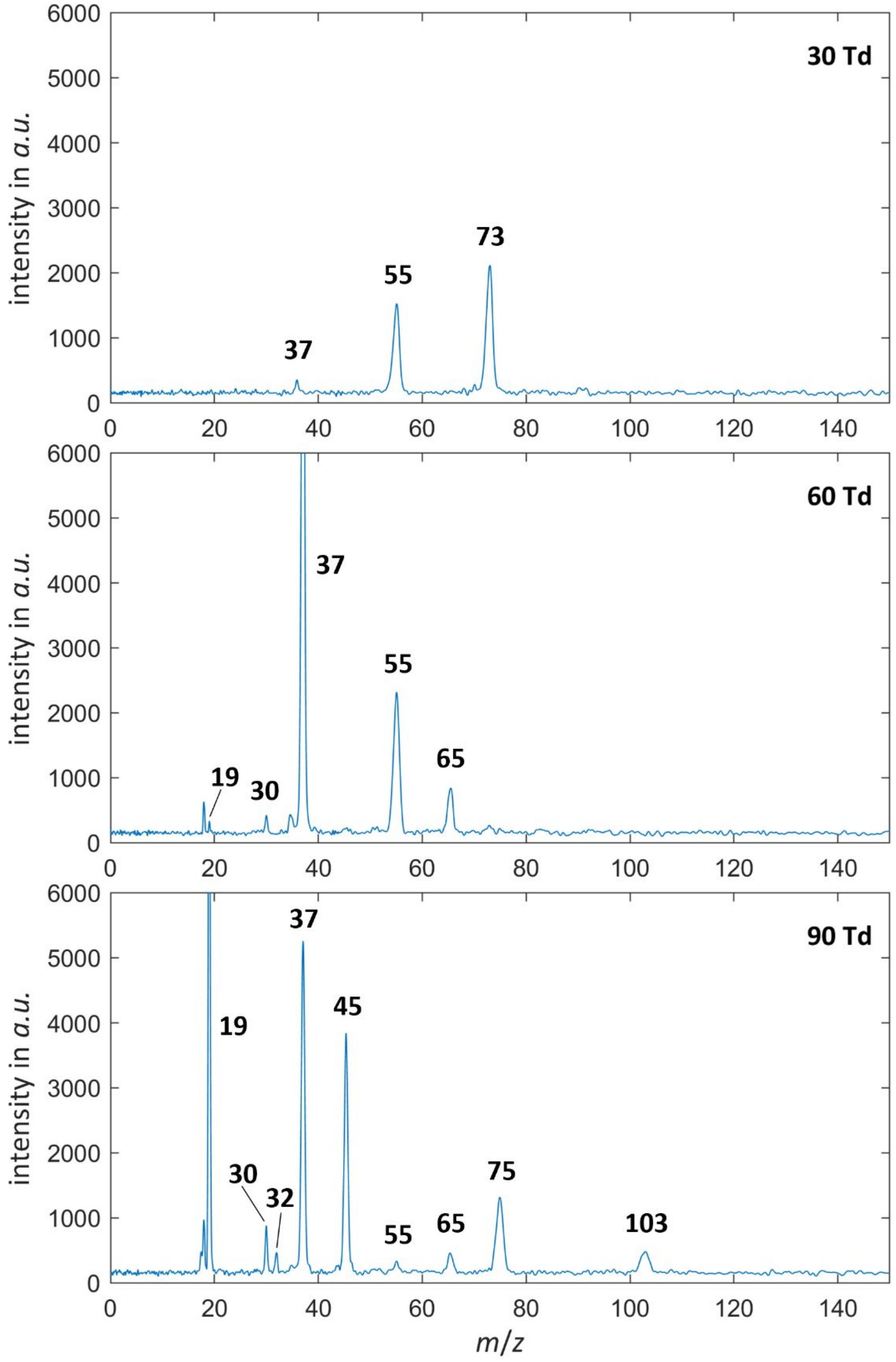

Figure S6: Mass spectra of the reactant ions, i.e., without any analyte. The reactant ions are composed of the protonated water clusters, $H^+(H_2O)_n$, at *m/z* 19, 37, 55, 73, as well as NO^+ at *m/z* 30, and O_2^+ a *m/z* 32. Note the background ions at *m/z* 75 and 103.

S3.2: 2-Propanol

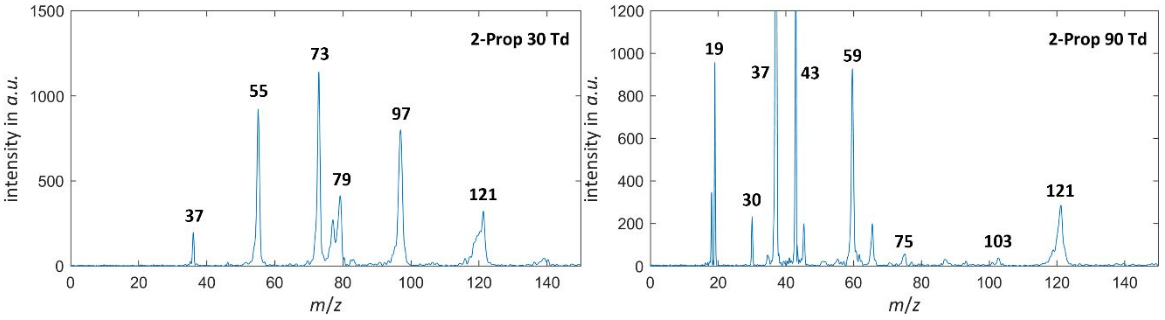

Figure S7: Mass spectra of protonated 2-propanol at 30 Td (left) and 90 Td (right).

Table S1: 2-Propanol ion species and their corresponding mass-to-charge ratios (*m*/*z*).

2-Propanol ion species	Mass-to-charge ratio (*m*/*z*)
R^+	43
ROH_2^+	61
$[ROH_2+H_2O]^+$	79
$[ROH_2+2(H_2O)]^+$	97
$[ROH_2+ROH]^+$	121

S3.3: 1-Butanol

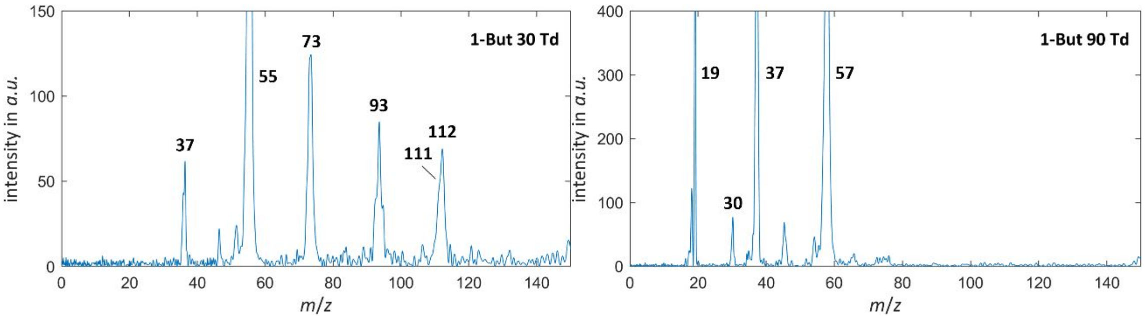

Figure S8: Mass spectra of protonated 1-butanol at 30 Td (left) and 90 Td (right).

Table S2: 1-Butanol ion species and their corresponding mass-to-charge ratios (*m*/*z*).

1-Butanol ion species	Mass-to-charge ratio (*m*/*z*)
R^+	57
ROH_2^+	75
$[ROH_2+H_2O]^+$	93
$[ROH_2+2(H_2O)]^+$	111

S3.4: 1-Pentanol

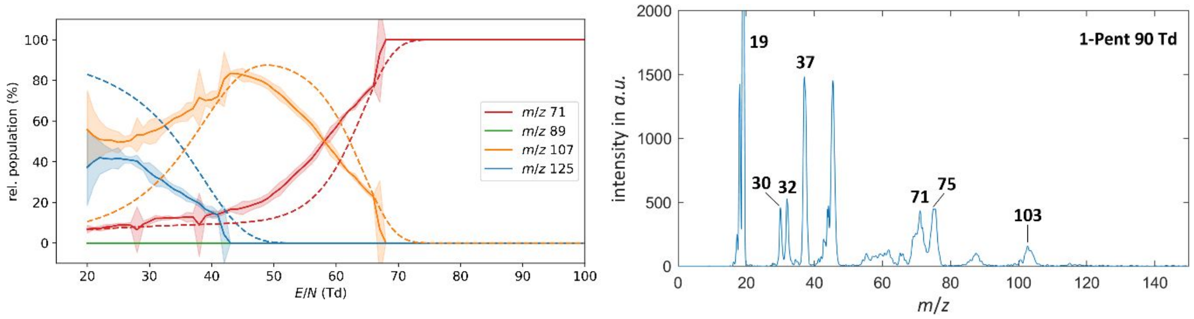

Figure S9: Measured (solid lines with error bars) and computed (dashed lines) ion populations of protonated 1-pentanol in the HiKE-IMS-MS over a reduced field strength range of 20 – 100 Td in the drift region (left) and mass spectra of protonated 1-pentanol at 90 Td (right).

Table S3: 1-Pentanol ion species and their corresponding mass-to-charge ratios (*m/z*).

1-Pentanol ion species	Mass-to-charge ratio (*m/z*)
R^+	71
ROH_2^+	89
$[ROH_2+H_2O]^+$	107
$[ROH_2+2(H_2O)]^+$	125

S3.5: 2-Hexanol

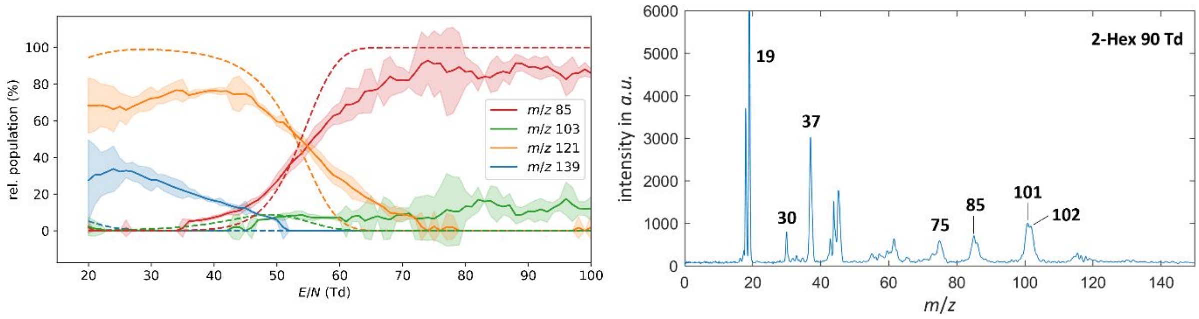

Figure S10: Measured (solid lines with error bars) and computed (dashed lines) ion populations of protonated 2-hexanol in the HiKE-IMS-MS over a reduced field strength range of 20 – 100 Td in the drift region (left) and mass spectra of protonated 2-hexanol at 90 Td (right).

Table S4: 2-Hexanol ion species and their corresponding mass-to-charge ratios (*m/z*).

2-Hexanol ion species	Mass-to-charge ratio (*m/z*)
R^+	85
ROH_2^+	103
$[ROH_2+H_2O]^+$	121
$[ROH_2+2(H_2O)]^+$	139

S4: Peak Position Analysis for 1- and 2-Butanol

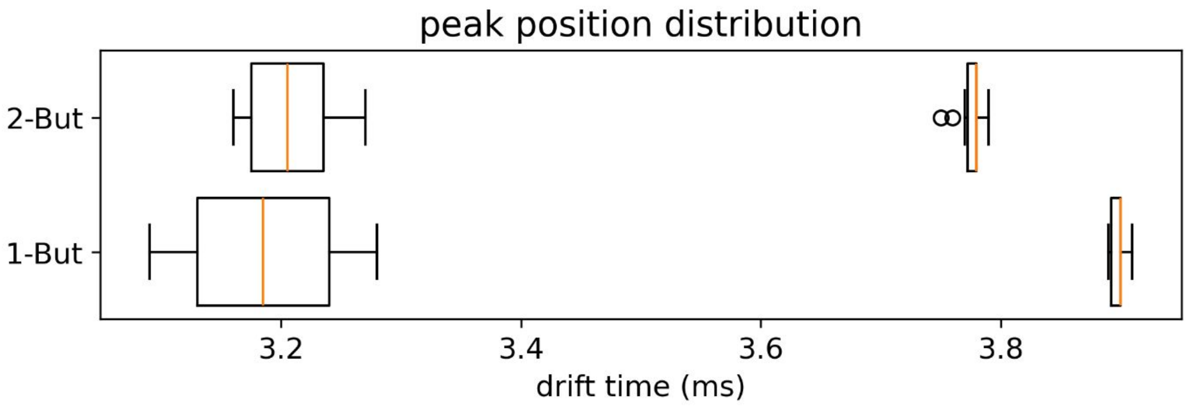

Figure S11: Distribution of the measured drift times for the precursor and fragment peak in the tandem IMS for both 1-butanol and 2-butanol for different RF_{pp} voltages.

S5: Modeling of the HiKE-IMS-MS data

Suppose we consider the following reaction system:

$$A \rightleftarrows B \rightarrow C$$

with respective rate coefficients k_{AB}, k_{BA}, and k_{BC}. The rate equations then read:

$$\frac{\mathrm{d}}{\mathrm{dt}}[A] = -\,k_{AB}[A] + k_{BA}[B] (S3)$$

$$\frac{\mathrm{d}}{\mathrm{dt}}[B] = +\,k_{AB}[A] - k_{BA}[B] - k_{BC} (S4)$$

$$\frac{\mathrm{d}}{\mathrm{dt}}[C] = +\,k_{BC}[B] (S5)$$

or:

$$\frac{\mathrm{d}}{\mathrm{d}t}\begin{pmatrix}[A]\\ [B]\\ [C]\end{pmatrix} = \begin{pmatrix}-\,k_{AB} & +\,k_{BA} & 0\\ +\,k_{AB} & -\,k_{BA}-k_{BC} & 0\\ 0 & +\,k_{BC} & 0\end{pmatrix} \cdot \begin{pmatrix}[A]\\ [B]\\ [C]\end{pmatrix} (S6)$$

where we call the matrix the rate coefficient matrix, $\boldsymbol{k}$. For this homogeneous, linear system of coupled differential equation, direct integration is possible:

$$\begin{pmatrix}[A]\\ [B]\\ [C]\end{pmatrix}_t = \exp(\boldsymbol{k}\cdot t)\cdot\begin{pmatrix}[A]\\ [B]\\ [C]\end{pmatrix}_{t=0} (S7)$$

To calculate the term $\exp(\boldsymbol{k}\cdot t)$ for a given $\boldsymbol{k}$, the reader is referred to Ref. 2.

In principle, we could simply equate the reaction time t with the drift time, t_D, as obtained from the mobility of the ions and the applied reduced field strength ($t_D = L/KE$). However, as the ion mobility changes during the drift due to the reaction involved, we perform a step-wise propagation with time steps between 10^{-8} and 10^{-6} seconds. Small time steps also help with the numerical stability of determining $\exp(\boldsymbol{k} \cdot t)$.

Thus, giving a starting condition, $\boldsymbol{P}(t=0) = \left(\begin{bmatrix} [A] \\ [B] \\ [C] \end{bmatrix}\right)_{t=0}$, we can propagate the ions through the drift tube in small time steps, Δt, and calculate the reaction turnover via $\exp(\boldsymbol{k} \cdot \Delta t)$. The propagation is terminated once the desired drift length has been reached:

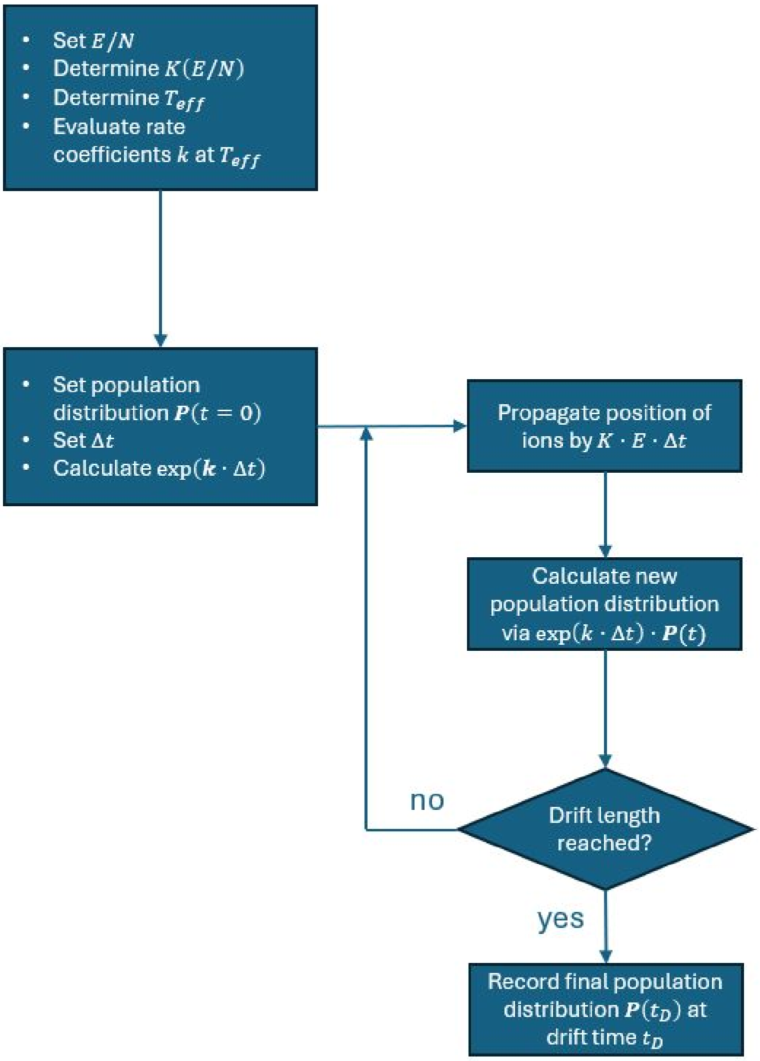

For more details, see Ref. 2,5.

References

(1) Allers, M.; Kirk, A. T.; Eckermann, M.; Schaefer, C.; Erdogdu, D.; Wissdorf, W.; Benter, T.; Zimmermann, S. Positive Reactant Ion Formation in High Kinetic Energy Ion Mobility Spectrometry (HiKE-IMS). *J. Am. Soc. Mass Spectrom.* **2020**, *31* (6), 1291–1301. DOI: 10.1021/jasms.0c00114. Published Online: May. 19, 2020.

(2) Haack, A.; Schaefer, C.; Zimmermann, S.; Hopkins, W. S. Validation of Field-Dependent Ion-Solvent Cluster Modeling via Direct Measurement of Cluster Size Distributions. *J. Am. Soc. Mass Spectrom.* **2023**, *34* (6), 1035–1046. DOI: 10.1021/jasms.3c00012. Published Online: Apr. 28, 2023.

(3) Kirk, A. T.; Grube, D.; Kobelt, T.; Wendt, C.; Zimmermann, S. High-Resolution High Kinetic Energy Ion Mobility Spectrometer Based on a Low-Discrimination Tristate Ion Shutter. *Anal. Chem.* **2018**, *90* (9), 5603–5611. DOI: 10.1021/acs.analchem.7b04586. Published Online: Apr. 16, 2018.

(4) Haack, A.; Ieritano, C.; Hopkins, W. S. MobCal-MPI 2.0: an accurate and parallelized package for calculating field-dependent collision cross sections and ion mobilities. *Analyst* **2023**, *148* (14), 3257–3273. DOI: 10.1039/D3AN00545C. Published Online: Jul. 10, 2023.

(5) Haack, A.; Schaefer, C.; Zimmermann, S. On the Arrival Time Distribution of Reacting Systems in Ion Mobility Spectrometry. *Anal. Chem.* **2024**, *96* (30), 12433–12443. DOI: 10.1021/acs.analchem.4c02010. Published Online: Jul. 15, 2024.
