# Supplementary figures and images for: Exploring Field-Induced Fragmentation of Protonated Alcohols: Mechanistic Insights and Stabilizing Ion–Solvent Clusters
Source: J Am Soc Mass Spectrom. 2025 Dec 15;37(1):310–20. doi: 10.1021/jasms.5c00348 (PMC12784389; doi:10.1021/jasms.5c00348)

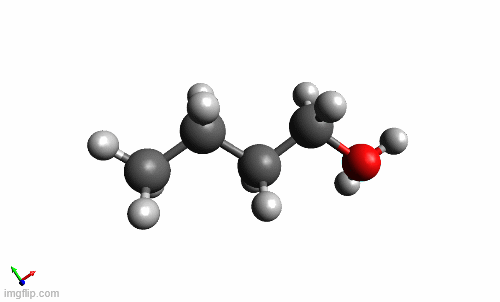

Supplement: Supplementary file 3 [file js5c00348_si_003.gif]
